# Quinine in Traumatic Fever

**Published:** 1843-10

**Authors:** 


*Quinine in Traumatic Fever.*—A case has lately occurred in the Hôpital des Enfans at Paris, in which Guersant has employed, with entire success, the sulphate of quinine in the treatment after amputation of both lower extremities. The patient, a poor boy, whose limbs had been frozen by exposure during a winter's night, and afterwards most injudiciously bathed in hot water, was brought to the hospital, the limb exhibiting a discolored spotty appearance, and other symptoms of incipient gangrene. This condition in six days, notwithstanding the application of bark-poultices and bottles of hot water to the extremities, so greatly increased as to render necessary an immediate amputation of both legs. The operation was successfully performed, and with little suffering to the patient, who gradually recovered, though not without indications of considerable fever and erysipelatous inflammation, both of which were subdued by doses of sulphate of quinine, amounting to twelve grains in a day. Guersant cites other cases, also, in which he has employed the same medicine with success, in all of which the leading feature was purulent reabsorption.—*London Lancet,* from *Gazette des Hôpitaux.*

